# Development of an *in vitro* pharmacokinetic/pharmacodynamic model in the presence of serum for studying micafungin activity against *Candida albicans:* a need for revision of CLSI susceptibility breakpoints

**DOI:** 10.1093/jac/dkad096

**Published:** 2023-04-18

**Authors:** Maria-Ioanna Beredaki, Maiken C Arendrup, David Andes, Joseph Meletiadis

**Affiliations:** Clinical Microbiology Laboratory, Attikon University Hospital, Medical School, National and Kapodistrian University of Athens, Rimini 1, Haidari 12462 Athens, Greece; Unit of Mycology, Statens Serum Institut, Copenhagen, Denmark; Department of Clinical Medicine, University of Copenhagen, Copenhagen, Denmark; Department of Clinical Microbiology, University of Copenhagen, Copenhagen, Denmark; Department of Medicine, University of Wisconsin, Madison, WI, USA; Clinical Microbiology Laboratory, Attikon University Hospital, Medical School, National and Kapodistrian University of Athens, Rimini 1, Haidari 12462 Athens, Greece; Department of Medical Microbiology and Infectious Diseases, Erasmus Medical Center, Rotterdam, The Netherlands

## Abstract

**Background:**

The CLSI breakpoint for micafungin and *Candida albicans* is 0.25 mg/L, higher than the CLSI epidemiological cut-off value (0.03 mg/L) whereas the EUCAST values are identical (0.016 mg/L). We developed a novel *in vitro* dialysis-diffusion pharmacokinetic/pharmacodynamic (PK/PD) model, confirmed correlation to *in vivo* outcome and studied micafungin pharmacodynamics against *Canida albicans*.

**Methods:**

Four *C. albicans* isolates, including a weak (F641L) and a strong (R647G) *fks1* mutants, were studied using a 10^4^ cfu/mL inoculum and RPMI medium with and without 10% pooled human serum. The exposure-effect relationship fAUC_0–24_/MIC was described for CLSI and EUCAST methodology. Monte Carlo simulation analysis included standard (100 mg i.v.) and higher (150–300 mg) doses q24h to determine the corresponding probability of target attainment (PTA).

**Results:**

The *in vitro* PK/PD targets for stasis/1-log kill were 36/57 *f*AUC_0–24_/MIC in absence and 2.8/9.2 *f*AUC_0–24_/MIC in the presence of serum, and similar for wild-type and *fks* mutant isolates. The PTAs for both PK/PD targets were high (>95%) for EUCAST susceptible isolates but not for CLSI susceptible non-wild-type isolates (CLSI MICs 0.06–0.25 mg/L). 300 mg q24h was needed to attain PK/PD targets for non-wild-type isolates with CLSI MICs 0.06–0.125 mg/L and EUCAST MICs 0.03–0.06 mg/L.

**Conclusion:**

The *in vitro* 1-log kill effect corresponded to stasis in animal model and mycological response in patients with invasive candidiasis, thereby validating the model for studying pharmacodynamics of echinocandins *in vitro*. EUCAST breakpoints were well supported by our findings but our data questions whether the current CLSI breakpoint, which is higher than the epidemiological cut-off values, is appropriate.

## Introduction

Echinocandins are front-line agents for the treatment of invasive candidiasis in both the neutropenic and non-neutropenic patients.^1^ Micafungin is the third echinocandin antifungal agents approved by the European Medicines Agency in 2008 for treatment of invasive candidiasis, oesophageal candidiasis and for prophylaxis in patients undergoing stem cell transplantation.^2^ It acts via non-competitive inhibition of the 1,3-β-D-glucan synthase that forms 1,3-β-D-glucan, an integral part of the fungal cell wall. Resistance is developed via mutations in two highly conserved hot-spot regions of the 1,3-β-D-glucan synthase gene *fks1* in *C. albicans* and most other *Candida* species and *fks1* and homologous regions of *fks2* genes in *Candida glabrata.*^3^

The minimal inhibitory concentrations of *fks* mutant isolates can be elevated a few (weak resistance) or several (strong resistance) dilutions above the epidemiological cut-off values (ECV/ECOFF). Although the CLSI epidemiological cut-off value 0.03 mg/L is close to the EUCAST ECOFF 0.016 mg/L, current susceptibility breakpoints of those two methodologies are notably different: *S* ≤ 0.25/R > 0.5 mg/L for CLSI and *S* ≤ 0.016/R > 0.03 mg/L for EUCAST. *In vitro* pharmacokinetic/pharmacodynamic (PK/PD) studies can be used to study micafungin pharmacodynamics at clinically relevant exposures and determine PK/PD breakpoints. Although, there are clinical and animal PK/PD studies,^4,5^ there are no validated *in vitro* PK/PD models for studying pharmacodynamics of echinocandins. Furthermore, echinocandin resistant *C. albicans* isolates have not been tested in PK/PD studies, as most previous studies were conducted in the era of susceptible isolates.

We therefore developed an *in vitro* PK/PD model that correlated with the *in vivo* activity of micafungin ^6^, explored the *in vitro* pharmacodynamics against susceptible and resistant *C. albicans* isolates, determined PK/PD breakpoints of EUCAST and CLSI and optimized micafungin dosing regimens for resistant isolates.

## Materials and methods

### *Candida* isolates and in vitro susceptibility

Two *fks* wild-type (WT) clinical *C. albicans* strains (CA580 and CA9817) previously used in animals^5^ with micafungin EUCAST/CLSI minimal inhibitory concentration (MIC) of 0.015/0.008 and 0.03/0.03 mg/L and two clinical *C. albicans* strains harbouring different *fks1* mutations (SSI-5318 strain with F641L *fk1* mutation, SSI-6683 strains with R647G *fk1* mutation) with micafungin EUCAST^7^/CLSI^8^ MICs of 0.03/0.06 and 0.5/0.5 mg/L, respectively, were used (Table 1).

**Table 1.** *In vitro* susceptibility of *C. albicans* isolates using CLSI M27 and EUCAST E. Def 7.3 methodology

Isolates	*fks*1 resistance mechanism	Median MIC (range) in mg/L	
		EUCAST	CLSI
*C. albicans* CA 580	WT	0.016 (0.008–0.016)	0.008 (0.008–0.015)
*C. albicans* CA 9817	WT	0.03 (0.016–0.03)	0.03 (0.03–0.06)
*C. albicans* CA SSI^a^-5318	F641L	0.03 (0.03)	0.06 (0.06)
*C. albicans* CA SSI^a^-6683	R647G	0.5 (0.25–0.5)	0.5 (0.5)

SSI; Staten Serum Institute

The isolates were stored in normal sterile saline with 10% glycerol at −70°C; 24 hours prior to study, the organisms were revived by subculturing on Sabouraud dextrose agar plates supplemented with gentamicin and chloramphenicol (SGC2, Biomerieux) to ensure purity and viability. Inoculum suspensions of the subcultured yeasts were prepared in sterile normal saline and adjusted after counting in a Neubauer hemacytometer to a final inoculum of 10^4^ cfu/mL. The CFU number was confirmed by quantitative cultures on SDA plates. All MIC testing was performed in triplicate with *Candida parapsilosis* ATCC 22019 and *Candida krusei* ATCC 6258 as quality control organisms to ensure reproducibility between tests.

### Antifungal drugs and medium

Micafungin (Astellas Pharma Inc.) was supplied as pure powder and used in the *in vitro* experiments. Stock solutions were prepared in sterile dimethyl sulfoxide (DMSO; CarloErbaReactifs-SDS, Val de Reuil, France) and stored at −70°C until use. RPMI 1640 medium with L-glutamine and without bicarbonate buffered to pH 7.0 with 0.165 M MOPS and supplemented with 100 mg/L chloramphenicol (AppliChem GmbH, Darmstadt, Germany) was used as the growth medium. Human serum was pooled from healthy volunteers and heat-inactivated at 56°C for 45 min. Briefly, whole-blood samples in silicon-coated tubes (BD Vacutainer^®^ tubes) were centrifuged at 4000×**g** for 10 min and supernatant serum was pooled in 50-mL Falcon tubes were then incubated in a pre-warmed water bath at 56°C for 45 min with constant swirling. Heat-inactivated human serum was aliquoted into Falcon tubes, stored at −70°C and used within 10 days after thawed and ketp at 4^o^C before testing. Because temperature and pH may influence protein binding, these factors were monitored throughout the *in vitro* experiments as an attempt to better mimic physiological conditions as closely as possible. Serum was diluted in RPMI medium to obtain 10% and 50% serum in RPMI 1 ×  medium.

### Static time-kill method

To study the pharmacodynamic differences between 10%, 50% and 100% serum, all four *C. albicans* isolates underwent static time-kill testing in 10%, 50% and 100% pooled human serum in CLSI RPMI medium containing 2-fold serial dilutions of micafungin ranging from 0.25 to 64 mg/L for 72 h at 37°C. The log_10_ cfu/mL were plotted against *tC*_max_/MICs for all concentration and strains. The *tC*_max_/MICs associated with 50% of maximal activity were determined using non-linear regression analysis based on Emax model as described next. Since no differences were found between 10%, 50% and 100% pooled human serum, 10% was used for all further experiments.

### In vitro PK/PD model

A previously developed two-compartment closed diffusion/dialysis *in vitro* PK/PD model was used^9^. Briefly, the model consists of an external compartment comprising a conical flask connected to a peristaltic pump and aninternal compartment (IC) of a 10 mL-volume semipermeable cellulose dialysis tube (Spectra/Por^®^ Float-A-Lyzer^®^ G2 with a molecular weight cut-off of 300 kDa; Spectrum Laboratories Inc., Breda, the Netherlands) inoculated with the conidial suspension (10^4^ cfu/mL). Post micafungin exposure growth outcomes were assessed in standard RPMI media and in RPMI media containing 10% pooled human serum. Repeated sampling of 100 μL was made from the IC to determine the drug concentration and samples were stored at −70°C until tested.

### In vitro pharmacokinetics

Different micafungin drug exposures with *C*_max_ ranging from 0.004 up to 32 mg/L and an average half-life of 15 h were simulated in the *in vitro* model in the presence and absence of 10% human serum. Drug concentrations were added at the corresponding *C*_max_ values in the *in vitro* model once daily. Micafungin levels were determined using a microbiological diffusion assay using an *Aspergillus fumigatus* strain (WT AZN8196 isolate, CLSI MEC 0.015 mg/L), as previously described.^10^ Drug concentrations correlated linearly with the diameter of zones of inhibited growth (*r*^2 ^= 0.98, *P* < 0.0001). The limit of detection (LOD) was 0.125 mg/L with interexperimental CV <3% for all drug concentrations tested. The time-concentration profiles were subjected to non-linear regression analysis based on the one-compartment model described by the equation *C*_t_ = *C*_o_*e*^−^*^k^*^/*t*^ where *C*_t_ (dependent variable) is the drug concentration at a given time *t* (independent variable), *C*_o_ is the initial drug concentration at 0 h, *e* is the physical constant 2.18 and *k* is the rate of drug removal. The half-life was calculated using the equation *t*_1/2 _= 0.693/*k* and compared with the respective values observed in humans. Finally, the AUC_0–24_ were calculated for each simulated dosage by applying the trapezoidal rule. For doses with concentrations lower than the LOD, AUC_0–24_ were estimated by linear extrapolation from the other doses.

### In vitro pharmacodynamics

The fungal load in the internal compartment was evaluated by 200-μL sampling at regular intervals up to 72 h, then 10-fold serially diluted in water and subcultured in SGC2 plates. Previous experiments showed that there was no carry over effect at high concentrations. Plates were incubated at 30°C for 24 h and colonies were counted at each dilution. Dilutions that yielded 10–50 colonies were used to determine the log_10_ cfu/mL at each timepoint. Time-kill curves were constructed by plotting log_10_ cfu/mL over time. Pharmacodynamic effects were assessed based on log_10_ cfu/mL reduction at 72 h compared to the start of therapy for each isolate and dosing regimen.

### PK/PD analysis

The PK/PD index *f*AUC_0–24_/MIC ratio was calculated for each simulated dose, isolate and experiment. The drug exposure-response relationship for up of 72 h of incubation, expressed with the log_10_ cfu/mL reduction at each dosing regimen and isolate compared to the start of therapy values versus *f*AUC_0–24_/MIC, was analysed in the presence and absence of 10% pooled serum by non-linear regression analysis based on the sigmoidal model with variable slope (*E*_max_ model) described by the equation *E* = *E*_max_ xEI^n^/(EI^n ^+ EI_50_^n^) where *E*_max_ is the maximum reduction in log_10_ cfu/mL, EI is the PK/PD index AUC_0–24_/MIC, EI_50_ is the AUC_0–24_/MIC required to achieve 50% of *E*_max_ and *n* is the the slope of the concentration-effect relationship (Hill coefficient). The goodness of fit of the *E*_max_ model was assessed by *R*^2^. Micafungin exposures associated with stasis (i.e. no log_10_CFU/mL reduction compared to the initial inoculum) and a 1-log kill (compared to the initial inoculum) effect were calculated and compared with the *in vivo* PK/PD targets previously found in a neutropenic murine model of candidiasis model after 4 days of treatment (12.7 *f*AUC/MIC for stasis, 25.2 *f*AUC/MIC for 1 log kill^5^ and clinical PK/PD target of 12.5–30 *f*AUC/MIC^4^). For calculation of *f*AUC/MIC in serum, a protein binding of 99.75% was taken into account. All data were analysed using the statistics software package GraphPad Prism, v.5.0, for Windows (GraphPad Software, San Diego, CA, USA). Two independent experiments were conducted.

### Monte Carlo simulations and analysis

To correlate the *in vitro* data with clinical outcome^4^, Monte Carlo simulation analysis was performed using the normal random number generator function of EXCEL spreadsheet (MS Office 2007) for 5000 patients infected with *C. albicans* isolates with micafungin MICs ranging from 0.008 to 8 mg/L and treated with the standard intravenous micafungin dosages of 100 mg IV once daily which corresponded to a total tAUC_0–24_of 97 ± 29 mg.h/L^11^. Higher doses of 150, 200 and 300 mg q24h resulting in tAUC_0–24_of 166 ± 39.9, 210 ± 69 and 338 ± 71 mg.h/L^11^ were simulated. The *f*AUC_0–24_ was calculated on the basis of the 0.25% unbound fraction of micafungin in human serum. Previously published MIC distribution data of *C. albicans* isolates with CLSI^12^ and EUCAST^13^ were used.

## Results

### Static time-kill method

**Figure 1.**
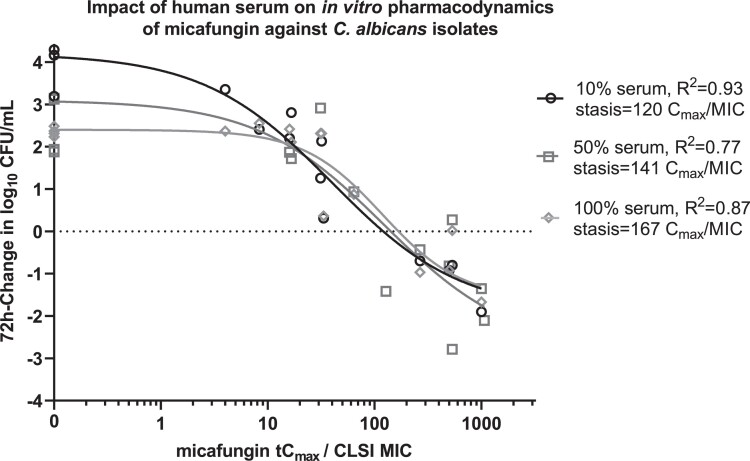
*In vitro* pharmacodynamics in 10%, 50% and 100% pooled human serum of micafungin against 3 *C. albicans* isolates tested in the static time-kill experiments.

Overall, the PK/PD index associated with a static effect were quite similar when performed in presence of 10%, 50% and 100% human pooled serum (120, 141 and 167 *tC*_max_/MIC, respectively), although differences in drug-free controls were noticed (Figure 1). Since no significant pharmacodynamic differences were found for *C. albicans,* further experiments were conducted in 10% human pooled serum.

### In vitro dynamic PK/PD model

**Figure 2.**
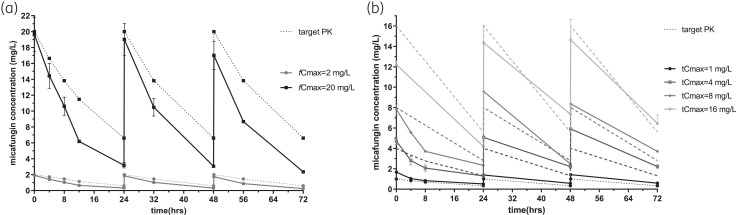
Representative time-concentration profile of simulated q24h micafungin dosing regimens in the *in vitro* PK/PD for *C. albicans* isolates, in the absence (a) and presence (b) of 10% human pooled serum. Data represent drug levels in the internal compartment of the *in vitro* model (solid lines) and the respective target values (broken lines). Error bars represent standard errors.

**Figure 3.**
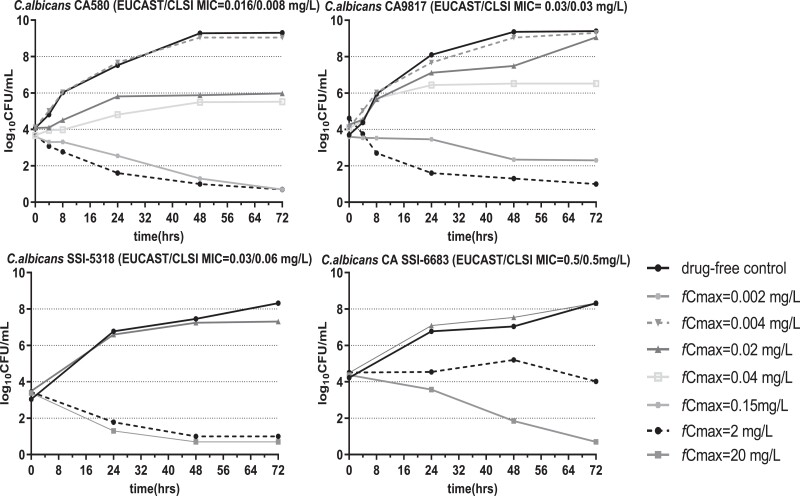
Time-kill curves in the *in vitro* PK/PD model for each simulated dosing regimens of micafungin against *C. albicans* isolates with increasing MICs using 10^4^ CFU/mL as the initial inoculum in the absence of 10% pooled human serum.

**Figure 4.**
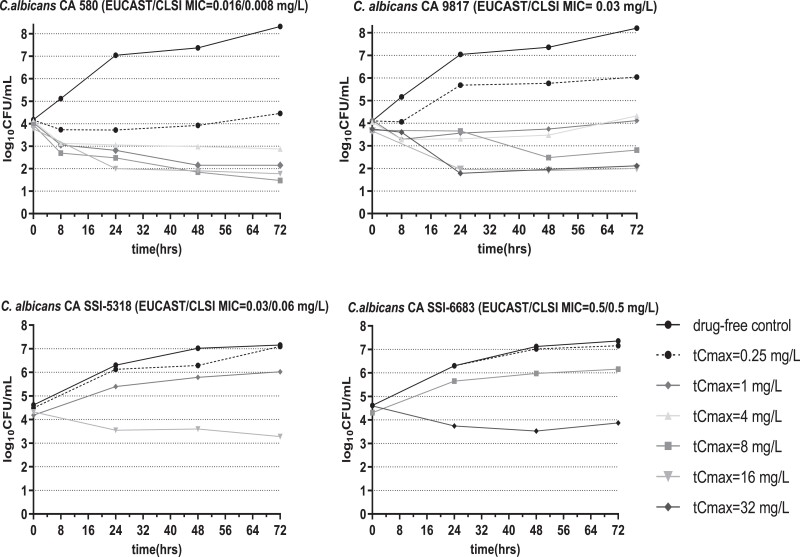
Time-kill curves in the *in vitro* PK/PD model for each simulated dosing regimens of micafungin against *C. albicans* isolates with increasing MICs using 10^4^ CFU/mL as the initial inoculum in the presence of 10% pooled human serum.

*In vitro* micafungin time-concentration profiles were closely matched to target pharmacokinetics, with an average *t*_1/2_ of 9 h (8–10 h) and 14 h (9–15 h) in the absence and presence of 10% pooled human serum, respectively (Figure 2). In absence of serum, *C. albicans* grew from a mean ± SD of 3.75 ± 0.41 log_10_cfu/mL at *t* = 0 h to 8.94 ± 0.53 log_10_ CFU/mL at *t* = 72 h in drug-free controls. Following micafungin exposure, a 0.5–3.6 log_10_cfu/mL decrease from the initial inoculum was observed at simulated exposures with *fC*_max _≥ 2 mg/L for all *C. albicans* isolates (Figure 3). In presence of serum, *C. albicans* grew from a mean ± SD of 4.37 ± 0.24 log_10_CFU/mL at *t* = 0 h to 7.76 ± 0.51 log_10_ cfu/mL at *t* = 72 h in drug-free controls, whereas a mean ± SD of 4.23 ± 2.74 log_10_cfu/mL reduction of fungal load, compared to that in drug-free controls, was observed in different micafungin exposures after 72 h, in line with *in vivo* findings.^5^ Micafungin produced a fungicidal effect (1–2log_10_ cfu/mL reductione from the initial inoculum) against *C. albicans* CA580 and CA9817 at *tC*_max_ ≥1 and ≥8 mg/L, respectively, while a small fungicidal effect was observed for *C. albicans* CA SSI-5318 and CA SSI-6683 at *tC*_max_ 16 and 32 mg/L, respectively (Figure 4).

### PK/PD analysis

**Figure 5.**
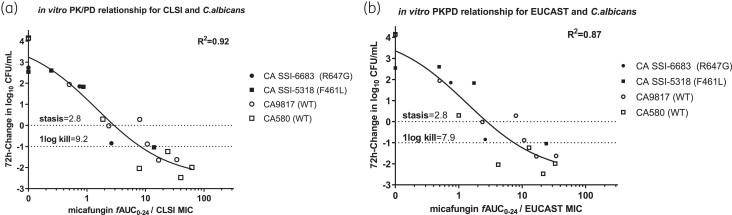
*In vitro* PK/PD relationship of micafungin for the *C. albicans* isolates tested in the *in vitro* PK/PD model using the 72-h change in log_10_CFU/mL versus *f*AUC_0–24_/MIC compared to the initial inoculum, in the presence of 10% human pooled serum for (a) the CLSI and (b) EUCAST methodology.

The *in vitro* PK/PD targets for CLSI are shown in Table 2. In the absence of serum, the *in vitro* PK/PD targets [mean (95%CI)] for stasis/1 log kill were 36/57 *f*AUC_0–24_/MIC for *C. albicans* isolates, while the presence of serum did have an impact on the *in vitro* exposure-response relationship, resulting in a 5–10-fold lower PK/PD target for stasis/1 log kill (mean 2.8/9.2 *f*AUC_0–24_/MIC). Importantly, none of the *in vitro* PK/PD targets in absence of serum matched the clinical (12.5 *f*AUC/MIC)^4^ or animal (12.7 *f*AUC/MIC for stasis, 25.2 *f*AUC/MIC for 1 log kill) ^5^ PKPD targets for *C. albicans* isolates, whereas in the presence of 10% serum, the *in vitro* 1 log kill PK/PD target of 9.2 *f*AUC/MIC was close to the clinical and static animal PKPD target for CLSI (Figure 5). The stasis and 1-log kill PK/PD targets for WT and mutant isolates were similar (2.9/9.1 for WT versus 3.1/9.4 for mutants, respectively using the same constrains in the *E*_max_ model).

**Table 2.** *In vitro* PK/PD relationship results of micafungin against *C. albicans* isolates for CLSI and EUCAST in the presence and absence of 10% human pooled serum

Conditions	Method	Mean (95%CI) *f*AUC_0–24_/MIC:	
		Stasis	1-log kill
10% human pooled serum	CLSI	2.8	9.2
		(1.8–4.6)	(5.2–18.7)
	EUCAST	2.8	7.9
		(1.6–4.8)	(4.3–17.5)
without 10% human pooled serum	CLSI	36	57
		(23–67)	(34–121)
	EUCAST	38	58
		(26–64)	(37–113)

For EUCAST, the *f*AUC_0–24_/MIC index required for stasis/1-log kill was similar, resulting in a mean (95% CI) of 2.8/7.9 *f*AUC_0–24_/MIC. The *in vitro* PK/PD target associated with stasis did not change significantly over time (2.2 versus 2.1 versus 2.8 *f*AUC/MIC for CLSI and 2.4 versus 2.1 versus 2.8 *f*AUC/MIC for EUCAST at 24, 48 and 72 h respectively).

### Bridging to human data

**Figure 6.**
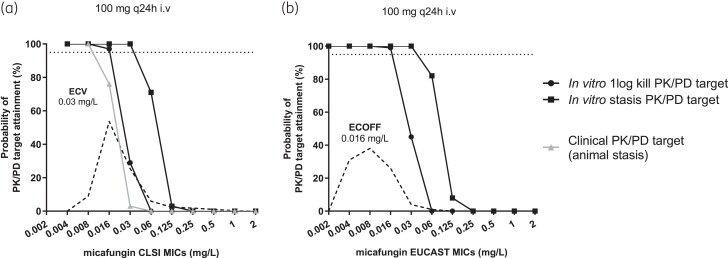
Probability of target attainment for the *C. albicans* isolates with increasing CLSI and EUCAST MICs for the standard 100 mg q24 dosing regimen of micafungin administered in patients, for the PD target associated with stasis and 1-log kill in the presence of 10% human pooled serum for the (a) CLSI and (b) EUCAST methodology, respectively. Horizontal lines correspond to 95% PTA.

**Figure 7.**
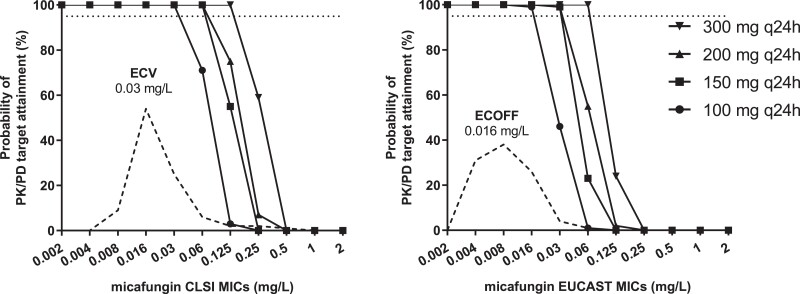
Probability of target attainment for *in vitro* stasis (CLSI) and 1-log kill (EUCAST) PK/PD target for *C. albicans* isolates with increasing MICs with the standard 100 mg q24 and higher (150, 200 and 300 mg 24 h) dosing regimen of micafungin. Horizontal lines correspond to 95% PTA.

The probability of target attainment for *in vitro* static, *in vitro* 1-log kill and clinical/animal static PK/PD targets with the standard dose of micafungin are shown in Figure 6. Similar PTAs were found for *in vitro* 1-log kill and clinical/animal stasis PK/PD CLSI targets further validating the *in vitro* PK/PD model. However, the PTA for both *in vitro* 1-log kill and clinical/animal stasis PK/PD CLSI targets were low for the CLSI WT population (CLSI MICs ≤0.03 mg/L) whereas the PTA for *in vitro* static target was >95% for the entire WT population. For EUCAST, the PTA for both *in vitro* static and 1-logkill PK/PD targets were >95% for the entire WT population (EUCAST MICs ≤0.016 mg/L) supporting current breakpoints of EUCAST. For isolates with low level resistance and CLSI MICs 0.06–0.125 mg/L and EUCAST MICs 0.03–0.06 mg/L higher doses (300 mg/d) will be needed to attain the *in vitro* PKPD targets (Figure 7).

## Discussion

An *in vitro* PK/PD model in the presence of 10% heat-inactivated pooled human serum was developed to explore *in vitro* micafungin pharmacodynamics against *C. albicans* isolates. *In vitro* 1-log kill effect (9.2 *f*AUC/MIC) was correlated with the PK/PD targets for mycological response in patients and for animal stasis (12.5 *f*AUC/MIC) validating the *in vitro* PK/PD model. Although EUCAST breakpoints (≤S/>R 0.016/0.016 mg/L with an area of technical uncertainty at 0.03 mg/L) were well supported, CLSI breakpoints (≤S/>R 0.25/0.5 mg/L) were questioned as neither *in vivo* (clinical/animal) nor *in vitro* PK/PD targets could be sufficiently attained for the susceptible but non-WT isolates in the MIC range 0.06–0.25 mg/L. Those breakpoints were determined collectively for *C. albicans*, *C. tropicalis* and *C. krusei* although their ECVs are markedly different (0.03, 0.06 and 0.125 mg/L, respectively) and manly based on the observation that most *fks* mutants at that time have MICs >0.25 mg/L whereas later studies detected mutants around ECV.^14,15^ No clinical data showed efficacy against non-WT *C. albicans* isolates with MICs 0.06–0.25 mg/L as almost all isolates had MICs <0.06 mg/L. However, *fks* mutant isolates are emerging despite candin therapy, which is regarded as an indirect indicator of insufficient infection control. The *in vitro* static PKPD CLSI target (2.8 *f*AUC/MIC) could be attained for the CLSI WT population with MIC  ≤ 0.03 mg/L indicating a revised CLSI S breakpoint of 0.03 mg/L should be considered. Higher doses of 150 and 300 mg q24h would be needed for isolates with low level resistance with CLSI MICs 0.06 and 0.125 mg/L and EUCAST MICs 0.03 and 0.06 mg/L, respectively, according to our results, but daily dosing >200 mg is not licensed for medical use.

Studying echinocandins’ pharmacodynamics is challenging because of the high protein binding. Serum does not only affect the pharmacokinetics but also the pharmacodynamics. Serum has been shown to increase micafungin MICs,^16,17^ even though this increase does not parallel the percentage of protein binding^17,18^ indicating increased antifungal activity of the predicted free drug concentrations in presense of serum. Similarly, another highly lipophilic drug posaconazole showed greater pharmacodynamic effect in human serum than predicted by the non-protein-bound serum concentration.^19^ Even though the effect of serum against the inhibitory activities of echinocandins against *Candida* spp. has been studied previously in static models,^17,18^ the effect on their antifungal activity in dynamic models is unknown. The PK/PD characteristics of micafungin may be altered in the presence of serum and could be of clinical relevance^20^ as found in the present study. Killing was enhanced in the *in vitro* PK/PD model in presence of human serum as the PK/PD target for 1-log kill in absence of serum was higher than in presence of serum 57 versus 9.2 *f*AUC_0–24_/MIC, respectively. The same phenomenon could be observed for other echinocandins as they also have higher protein binding rates and therefore their activity merit revaluation in presence of serum. Apart from the lower growth of drug-free control, no significant differences on the *in vitro* activity were found in the static model between 10%, 50% and 100% serum as static and maximum killing effects were similar indicating that even 10% serum is sufficient to exert serum effect on micafungin activity. Such differences in drug-free control was found also in the *in vitro* dynamic model, without affecting micafungin activity. The *f*AUC/MIC targets were similar for susceptible and resistant isolates as found previously in animal studies for *C. glabrata* WT and mutants.^21^

In addition, the *in vitro* PK/PD target for 1-log kill in presence of serum was close to the clinical PK/PD target for mycological response (5000 tAUC_0–24_/MIC)^4^ and to the animal PK/PD target for stasis (5.299 tAUC_0–24_/MIC i.e. 12.5 *f*AUC_0–24_/MIC).^14^ To our knowledge, this is the first validated model for studying pharmacodynamics of echinocandins. The echinocandins are extensively bound to serum proteins with rates of 98% reported for caspofungin, 99.75% for micafungin and >99% for anidulafungin.^22^ According to the free drug hypothesis, only the unbound compound can take part in antifungal activity, meaning that for micafungin, only 0.25% is thought to be involved in antifungal activity. However, it has be shown that the free drug hypothesis seems to be unsuitable to apply to the pharmacodynamics of micafungin, underestimating its antifungal activity.^17,18,4^ The previously mentioned facts gave ground to the use of serum in the *in vitro* diffusion-dialysis model used in our study. A potential explanation for this increasing killing in presence of human serum could be the restricted growth in serum or enhanced distribution of drug from serum proteins to lipophilic fungal cell membrane. It was indeed previously found that albumin acts as a potential carrier molecule to facilitate antifungal drug delivery to *Aspergillus* hyphae.^4^

Monte Carlo analysis based on the CLSI PK/PD targets for *in vitro* 1-log kill, mycological response in patients with candidemia/invasive candidiasis and animal stasis resulted in similar PTA validating further the *in vitro* PKPD model. However, the PTAs for those PK/PD targets were low for CLSI non-WT susceptible isolates with MICs 0.06–0.25 mg/L questioning the current CLSI clinical breakpoints (*S* ≤ 0.25/*R* > 0.5 mg/L). Furthermore, on the basis of those PK/PD targets, the standard dosing of micafungin is hardly sufficient for WT *C. albicans* isolates with CLSI MIC values of 0.03 mg/L as found previously in multiple publications,^23–26^ despite the evidences of clinical efficacy against WT isolates questioning the validity of those PK/PD targets. The clinical PK/PD target was determined on the basis of the data of phase 3 clinical studies with patients with invasive candidiasis/candidemia all of which were infected with WT *C. albicans* with CLSI MICs ≤ 0.016 mg/L, and thus the study was not powered to find PK/PD targets associated with failure (a common problem of such analyses where isolates with high MICs and patients with low exposures are needed).^4^ Furthermore, animal PK/PD targets were determined based on drug exposure in serum and fungal burden in kidneys of neutropenic animals and therefore they may not describe PK/PD characteristics in patients with candidemia.^27^ In absence of an alternative PK/PD target, this discrepancy remained unresolved. In this study, the *in vitro* PK/PD target for stasis provides an alternative endpoint that can support a CLSI susceptibility breakpoint at the ECV of 0.03 mg/L, thus covering the entire WT population with the standard dose of micafungin (100 mg 24 h) in agreement with what we found for the EUCAST data. A higher dose would probably be needed for *fks* mutants that confer weak resistance resulting in elevated MICs up to 0.06 mg/L.^28^

This study is associated with limitations. Although we included WT as well as low and high MIC *fks* mutant isolates, the number of isolates was low. However, PK/PD targets were similar for WT and *fks* mutants as previously found in neutropenic animal models for *C. glabrata*.^21^ A second limitation is that our *in vitro* model does not take fungal virulence or host immunity into account. *fks* mutants may be less virulent than WT isolates,^29^ and micafungin possesses immunomodulatory effects.^30^ It is possible that these factors may potentiate micafungin activity and therefore the findings mainly apply only to neutropenic patients and possibly to non-neutropenic patients for species with low virulence.

In conclusion, an *in vitro* PKPD model that correlates with *in vivo* outcome has been developed for studying pharmacodynamics of echinocandins against *Candida* spp. The *in vitro* 1-log kill effect corresponded to stasis in animal model and mycological response in patients with invasive candidiasis. EUCAST breakpoints were well supported by current findings but our data suggest that the CLSI S breakpoint for micafungin and *C. albicans* should be lowered from 0.25 mg/L to the ECV 0.03 mg/L. The same reduction may be needed also for micafungin and other *Candida* species and for anidulafungin as S breakpoints are higher than the corresponding ECVs although the differences are smaller (one 2-fold dilution) than the difference for micafungin and *C. albicans*. Isolates with weak *fks* mutations that confer low level resistance may be treated with higher doses micafungin, a hypothesis that needs clinical investigation.
